# Quality of Life and Breast Cancer: How Can Mind–Body Exercise Therapies Help? An Overview Study

**DOI:** 10.3390/sports5040079

**Published:** 2017-10-13

**Authors:** Anne Marie Lunde Husebø, Tormod Lunde Husebø

**Affiliations:** 1 Faculty of Health Sciences, University of Stavanger, Kjell Arholmsgate 41, 4036 Stavanger, Norway; 2 Research Department, Stavanger University Hospital, Armauer Hansens vei 2, 4011 Stavanger, Norway; 3 Faculty of Arts and Education, University of Stavanger, Telegrafdirektør Heftyes vei 35, 4021 Stavanger, Norway; tormod.lunde.husebo@gmail.com

**Keywords:** breast cancer, complementary therapy, mind–body exercise, overview, qigong, quality of life, tai chi chuan, yoga

## Abstract

Breast cancer survivors experience extensive treatments, threatening their quality of life. Complementary therapies used as a supplement to cancer treatment may control symptoms, enhance quality of life, and contribute to overall patient care. Mind–body exercise therapies might motivate cancer survivors to exercise, and assist them in regaining health. The purpose of this overview study is to study benefits from mind–body exercise of yoga, tai chi chuan and qigong upon quality of life in breast cancer populations. A systematic overview of reviews was applied. Literature search in five electronic databases and in reference lists was performed during April 2017. In addition, experts in the field were consulted. Of 38 identified titles, 11 review articles, including six meta-analyses were found eligible for review. Methodological quality was high for the majority of quality domains. Yoga, the most studied mind–body therapy, was found to benefit breast cancer patients’ psychological quality of life, while less support was established concerning physical quality of life elements. The evidence of improvements of quality of life from tai chi chuan and qigong remains unclear. Breast cancer survivors’ experiences of psychological and social well-being may be enhanced by practicing yoga.

## 1. Introduction

This overview of research literature presents results from complementary therapies including bodily movement interventions of yoga, tai chi chuan, and qigong (i.e., mind–body exercise) offered to breast cancer survivors as a supplement to traditional cancer treatment with the intention of improved quality of life (QoL). Breast cancer requires prolonged and extensive therapy, which may reduce the patient’s quality of life and sense of control [1]. Women with breast cancer experience issues related to an altered body image which may affect self-esteem and self-image, and pose a challenge for their QoL [2].

According to Deng and Cassileth [3], complementary therapies in cancer care are used to supplement regular cancer care, such as supportive measures to control symptoms, enhance QoL and well-being, and contribute to overall patient care. Horneber et al. [4] argued that the use of complementary therapies among cancer survivors has increased significantly, from 25% in the 1970–1980s to 49% since 2000, suggesting that today’s cancer survivors are increasingly interested in supplementing medical treatment with unconventional therapies, methods and natural products. Researchers claim that health care services should develop strategies to satisfy the patient’s knowledge and desire for the use of complementary therapies, and that more research is needed to evaluate the efficacy of such therapies [4].

There are a vast variety of complementary therapeutic interventions, including natural medicine, self-help techniques and various forms of physical training. In the United States, breathing exercises, meditation, massage and yoga are among the most commonly used complementary treatments in the general population, regardless of the participants’ health condition [5].

Acknowledged mind–body exercise therapies are yoga, tai chi chuan, qigong, music therapy, arts therapy, massage, acupuncture, meditation, mindfulness and visualization. All of these intend to boost the brain’s capacity to positively affect bodily functions and symptoms, thereby reducing anxiety, depression and stress, and managing pain, nausea, breathing difficulties and fatigue [6]. An important question of interest to researchers, cancer survivors and clinicians is whether such complementary therapies are effective. Research on complementary therapies is growing rapidly and well-established research centers worldwide (e.g., Cochrane Library, Natural Medicines) provide high-quality pooled evidence of the effect on patient outcomes. In the wake of this, overviews of review literature have emerged. Lee et al. [7] performed an overview study of research on the effectiveness of qigong on any health condition and patient outcome, stating that a conclusion concerning the effect from qigong was not possible due to the poor research quality of qigong trials. McCall et al. [8] conducted an overview to investigate the characteristics and effects of yoga interventions on chronic health conditions, concluding that yoga may reduce symptoms of anxiety and depression. Kelley and Kelley [9] performed a meta-analysis of systematic reviews to investigate the effects of different complementary therapies in vast patient populations, finding that yoga exercise had a positive effect on QoL. To our knowledge, our study is the first overview of literature focusing on the benefits from mind–body exercise on QoL in breast cancer populations. 

This paper attempts to evaluate critically the quality of research on complementary mind–body therapies that apply bodily movement among breast cancer survivors, and to summarize, compare and discuss their conclusions. It will in particular attempt to answer the following research question: How beneficial are the exercise of yoga, tai chi chuan and qigong upon QoL in breast cancer populations?

## 2. Materials and Methods

To synthesize evidence, a systematic overview was performed of multiple literature reviews, systematic reviews and meta-analyses. The methodology for an overview of reviews is similar to that of a systematic review, and is appropriate when relevant systematic reviews are obtainable [10,11]. In the current overview, the framework by Smith et al. [12] guided the identification and selection of eligible review studies, followed by summarization and discussion of conclusions.

### 2.1. Data Sources and Searches

The search for relevant literature took place in April 2017 in the electronic databases Cumulative Index of Nursing and Allied Health (CINAHL), Medline, Academic Search Premier and Sport Discus (Elton B. Stevens Company (EBSCO) databases). Additionally, a parallel search was conducted in the Cochrane Library. To assure accuracy, the literature search was performed in duplicate by the first author and a university librarian. The search comprised systematic reviews and meta-analyses published in English between 2007 and 2017, that contained specific search terms in the title or abstract. Broad search terms applied were breast cancer, review, quality of life, yoga, tai chi chuan and qigong. A secondary, manual search was performed by screening the reference lists of the included reviews and meta-analyses. Finally, experts in the field were consulted for possible eligible articles. Our search strategy is illustrated in Table 1.

### 2.2. Study Selection and Inclusion Criteria

Study selection followed the PRISMA (Preferred Reporting Items for Systematic Reviews and Meta-Analyses) [13] guidelines. To detect relevant studies, titles and abstracts were assessed for relevance and duplication, based on the established inclusion criteria of: (a) literature review, systematic reviews and/or meta-analysis; (b) interventions testing the efficiency of mind–body therapies that included an element of bodily movement (i.e., yoga, tai chi chuan, qigong); (c) patient outcome of QoL; (d) populations of breast cancer survivors during treatment and recovery. Eligible abstracts were retrieved in full-text copies for further examination, to establish relevance to the research question before final inclusion and data extraction [12]. 

### 2.3. Data Extraction and Quality Assessment

The review team (A.M.L.H., T.L.H.) thoroughly read and assessed full-text versions of the included papers, and extracted information on study characteristics (author, publication year, number of included studies, cancer population, intervention category and contents, patient outcome and effect). Assessment of the reviews’ methodological quality was performed in duplicate (A.M.L.H., T.H.L.) by using the tool “A MeaSurement Tool to Assess systematic Reviews” (AMSTAR), developed by Shea et al. [14] for quality assessment of multiple systematic reviews. The following 11 domains were rated: review design, literature search, data extractor involvement, status of publication (i.e., grey literature), list of included and excluded studies, characteristics of included studies, documentation of quality assessment, use of quality assessment in concluding remarks, methods of synthesis of findings, assessment of publication bias, and statement of conflict of interest [14]. Each domain achieved either “Yes” (1 point), “No” (0 points), or Can’t tell (CA)” (0 points), and each study could achieve a maximum quality score of 11 points. AMSTAR scores ≥ 10 were considered ‘very high’, 8–9.9 scores as ‘high’, 4–7.9 scores as ‘medium’, and 0–3.3 scores as ‘low’ quality [8].

## 3. Results

**Figure 1:**
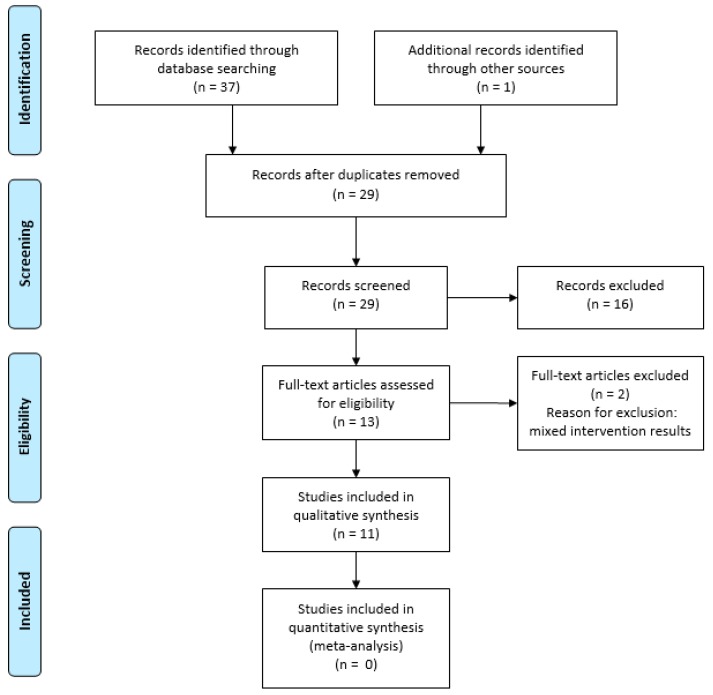
Preferred Reporting Items for Systematic Reviews and Meta-Analyses (PRISMA) flow chart of the identification and selection process (Moher et al., 2009).

The literature search yielded 38 titles related to reviews on the effects of mind–body interventions. After duplicates removal and screening of 28 abstracts, 12 full-text review articles were assessed for eligibility. Two full-text articles were excluded due to reporting of results which made it difficult to interpret effects from yoga. Finally, 11 review articles of which six comprised a meta-analysis were found eligible for inclusion in the overview study. The study selection is illustrated in a PRISMA flow diagram (Figure 1).

### 3.1. Characteristics of Mind–Body Therapy Studies

The included reviews were published between 2007 and 2017. The studies comprised patients both during and after primary treatment. Shneerson et al. [15] and Stan et al. [16] studied effects from interventions after cancer treatment. Lee et al. [17], Pan et al. [18], Pan et al. [19] and Zhang et al. [20] included participants who were undergoing cancer treatment. The five remaining studies researched the effects of interventions both during and after treatment [21,22,23,24,25]. The populations in the original studies were mainly women with breast cancer, aged 18 to 70 years. Buffart et al. [22] and Shneerson et al. [15] also studied the effect of mind–body interventions among other cancer sites, in one of thirteen, and three of thirteen original studies, respectively. 

Concerning mind–body interventions, eight of the eleven review studies included yoga [15,16,18,20,21,22,23,24,25], and in five of these, yoga was tested as a sole mind–body therapy [18,20,22,23,24,25]. Tai chi chuan was studied in three of the reviews. Two reviews included solely tai chi chuan exercises [17,19], while Stan et al. [16] included tai chi chuan with yoga, pilates and qigong. Qigong was studied in two reviews. Shneerson et al. [15] included one qigong intervention along with four yoga interventions, while Bleakely and Stinson [21] reported from one qigong intervention and four yoga interventions. 

Eight of the included reviews were characterized as systematic [15,17,18,19,20,22,23,24], and six of these were meta-analyses aiming at studying effects from complementary therapies on patient outcomes of quality of life (QoL) [15,18,19,20,22,23]. Eight articles reviewed only randomized controlled designs [15,18,19,20,21,22,23,24], while three also included studies with other quantitative designs [16,17,25]. One of the randomized, controlled trials (RCT) in Levine and Balk [25] also comprised qualitative data collection. Between eight and thirty-five original studies were included in the review articles. Effect on global QoL was reported in five of the review articles [15,19,21,24,25], and health related quality of life (HRQoL) was reported in 4 of the included studies [17,18,22,23]. Shneerson et al. [15] reported an effect on physical and psychological QoL. Buffart et al. [22] reported effects of complementary therapies on general QoL. Only Levine and Balk [25] reported about disease-specific quality of life. The extracted information is included in a summary table (Table 2).

### 3.2. Methodological Quality of Included Reviews

Appraisal of the methodological quality using the AMSTAR instrument (Table 3) revealed an AMSTAR median score of eight across included studies (range: 3–9). Seven of the eleven reviews achieved a high-quality score (AMSTAR score: 8–9.9), considered to satisfy at least eight of eleven domains (>70%) [15,18,19,20,22,23,24]. Three of the reviews returned medium-quality scores (AMSTAR score: 4–7.9) [16,17,21], and one review was of low methodological quality (AMSTAR score: 0–3.9) [25]. The reviews were published within a time span of seven years (2011–2017). No differences in methodological quality were identified relating to early or later time of publishing. 

Across included reviews, three domains stood out as poorly addressed. The quality domain of (4) “status of publication (i.e., grey literature) as an inclusion criteria” was addressed in three of the ten reviews [16,24]. A list of included and excluded studies (domain 5) was not provided by any of the reviews, while two studies addressed the domain (10) “likelihood of publication bias assessed”, stating that risk of publication bias was assessed by use of funnel plots [15,23]. Domains (1) and (3) were met in all studies, while ten of the eleven reviews reported on duplicate study selection and data extraction (domain 2) [15,16,17,18,19,20,21,22,23,24], and provided a list of the characteristics of the included studies (domain 6) [15,16,17,18,19,20,21,22,23,24].

Concerning heterogeneity in the six meta-analyses, all studies calculated the presence of heterogeneity. All studies but Buffart et al. [22] displayed heterogeneity information in forest plots. Significant heterogeneity was reported and discussed in five of the meta-analyses [18,19,20,22,23]. In Shneerson et al. [15], results from heterogeneity testing was displayed by Forest Plot for one of four QoL outcomes, and reported as not applicable in three of the rest of the outcomes. This finding was not discussed further in the paper. 

### 3.3. Program Features and Adherence

The majority of the reviews evaluating yoga as a mind–body therapy reported the applied yoga styles as Integrative (i.e., physical poses, breathing techniques and meditation), Iyengar yoga or Hatha yoga. The studies of Levine and Blak [25], Shneerson et al. [15] and Stan et al. [16] also reported on Restorative yoga (i.e., passive stretching). All of the included reviews provided information on frequency (times/week) and length (weeks) of the yoga programs. Pooled results from the reviews show that the programs were performed 1–3 times per week, and over a range of 3 weeks to 6 months. All reviews reported the duration (minutes/session) of the yoga sessions/classes, showing a variation of 30–120 min. 

Tai chi chuan interventions were reported in three reviews [16,17,19]. Across reviews, the tai chi chuan programs were performed for 50–60 min, 1–3 times per week over 6–24 weeks. Tai chi chuan styles were reported in two reviews. In Pan et al. [19], the majority of the trials applied the styles of Chi Kung and Yang, while in Stan et al. [16], all five included tai chi chuan trials that applied Yang style.

Two of the reviews included studies applying qigong as a mind–body therapy. Shneerson et al. [15] included one original study on medical qigong, practiced for 90 min, 2 times per week for 10 weeks. Stan et al. [16] included two qigong intervention studies that used a variety of external, medical and chan-chuang qigong.

Program adherence was reported in four of eleven reviews. Buffart et al. [22] found attendance to yoga classes varied from 58 to 88%, with inconsistent results concerning the association between yoga class adherence and quality of life. In the discussion of results, Stan et al. [16] reported excellent program evaluation concerning adherence to yoga in the reviewed studies, and claimed yoga interventions were popular among women with breast cancer. In the review by Harder et al. [24], adherence was reported in five of eighteen yoga interventions, showing a heterogeneous picture of class attendance. Here, a neutral or positive effect from yoga on QoL outcomes was associated with adherence to the program. Two of the reviews reported attrition as a risk of bias. In Cramer et al. [23], four of twelve included trials had high drop-out rates, while half the studies in the meta-analyses of Zhang et al. [20] reported a large amount of attrition. None of the reviews that focused specifically on benefits from tai chi chuan [17,19] reported adherence rates from the included trials.

### 3.4. Effect of Mind–Body Therapies on Quality of Life

Of the nine reviews on yoga interventions, all showed improvements in psychological QoL components, while physical components remained unchanged in the majority of the reviews. Five reviews on yoga included a meta-analysis, and established effects sizes ranging from a low of 0.27 (small effect) to a high of 0.85 (large effect) [15,18,20,22,23]. Among reviews without meta-analysis, Bleakley and Stinson [21] concluded that in half the studies of mind–body therapies, yoga had an effect on global QoL. Harder et al. [24] established that yoga showed moderate-to-large effects on global and emotional QoL, but had no effect on physical QoL. Levine and Balk [25] identified significant benefits from yoga on breast cancer patients’ physical well-being, emotional health, social functioning and functional adaption. Stan et al. [16] found positive and statistically significant, or trending toward significance, effects of QoL from yoga. Improvements from yoga seemed to be related to the amount of yoga and the intervention duration. A greater number of yoga classes were found to significantly correlate with improvements in QoL [24], while yoga interventions of more than three months duration had the largest effects on QoL [18]. None of the reviews distinguished between different yoga styles regarding the effect on QoL.

Little evidence was found from the overview that tai chi chuan has effect on QoL for breast cancer survivors. Pooled results in one tai chi chuan meta-analysis suggested no significant improvement in psychosomatic (*p* = 0.07), social (*p* = 0.44) and emotional well-being (*p* = 0.46), or in global QoL (*p* = 0.61) [19]. Lee et al. [17] reported inconclusive results from four tai chi chuan interventions. Positive inter-group differences (*p* < 0.01) were found for HRQoL and self-esteem in one RCT, and for global QoL (*p* < 0.01) in one non-randomized controlled study. In Stan et al. [16], improvements from tai chi chuan on QoL were studied in two RCTs. One reported positive change in HRQoL domains of physical functioning and mental health, and one found improved global QoL after 12 weeks of performing tai chi chuan.

Qigong was studied in two of the reviews, revealing no statistically significant improvement on QoL components. Shneerson et al. [15] found uncertain effects from qigong, based on only one study. In Stan et al. [16], QoL outcomes were studied in two of four qigong trials, and qigong was found efficient for improvements of QoL in one RCT. In this RCT only 34% of the participants were breast cancer survivors. 

## 4. Discussion

The primary goal of this overview study was to investigate effects from mind–body therapies of yoga, tai chi chuan and qigong on QoL in breast cancer survivors, and discuss how these therapies could support cancer survivors through treatment and recovery. The current overview indicates that yoga as a complementary therapy seems well documented as effective for QoL among women with breast cancer. Practicing yoga may in general be positive for both global and health related QoL. The results concerning effects from tai chi chuan and qigong are less promising, as evidence on positive change in QoL was not established.

The rationale for including exercise as complementary to cancer treatment is built on the understanding of the concept of treatment as something more than simply curing disease. Complementary bodily movement therapies might motivate cancer survivors to exercise, and assist them in developing strategies for coping with stress and regaining health [26]. The overview suggests that breast cancer survivors are most likely to experience positive change in the psychological components of QoL from practicing yoga, like improved self-esteem and body image, stress reduction, increased sleep quality, less anxiety and depression, and better emotional and social well-being [15,16,18,20,23,24,25]. Similar results have been found by McCall et al. [8] and Pascoe and Bauer [27], stating that yoga may decrease anxiety and depression. Moreover, a pooled result from five meta-analyses on yoga trials among women with breast cancer yielded statistically significant moderate-to-large effects on psychosocial QoL outcomes [9]. Explanations as to why yoga exercises positively influence the psychosocial side of QoL might be found in the nature of yoga and its union of body, mind and spirit. Daley [28] claimed that exercise is a form of behavioral activation, leading to improvement of the patient’s self-esteem and mastery experience towards recovery. Such psychological benefits from exercise may be underestimated by patients and health professionals. Exercise professionals working in cancer care and rehabilitation should consider promotion of yoga as strengthening the psychological QoL in breast cancer survivors.

Positive effects from yoga on the physical well-being components of QoL were less prominent in our overview study, and only the review by Levine and Balk [25] reported significant effects from yoga on physical well-being components of symptom distress and treatment side-effects. Based on their findings, practicing yoga may relieve chemotherapy-induced symptoms of nausea and vomiting, and increase the patient’s ability to cope with her physical condition. Yoga styles reported in the included reviews were derived from Hatha yoga, which combine postures, breathing and meditation. Although considered a more physical yoga style, Hatha yoga is a low impact physical exercise [29]. Moreover, ranging from 2 to 4 metabolic equivalents (METS) (1 MET = the amount of oxygen consumed while resting), yoga does not meet recommendations for improving or maintaining cardiovascular fitness [30]. Although yoga has sparse effects on muscle strength, it may improve balance and mobility. This is an important aspect related to common side effects from breast cancer treatment that causes stiffness and postural instability [31]. Yagli et al. [32] suggested yoga as an adjunct to aerobic exercise programs for breast cancer survivors, to obtain both physical and psychosocial wellness.

Regarding the mind–body therapies of tai chi chuan and qigong, the overview shows that they have uncertain effects on quality of life in women with breast cancer. One explanation can be the limited number of tai chi chuan and qigong interventions included in the reviews, which limits the ability to draw firm overview conclusions on effectiveness, and generalize findings. Publication bias was not addressed in the current reviews, and cannot be ruled out as a source to lack of available tai chi chuan and qigong intervention research. Lee et al. [7] claim that in addition to inadequately documented effects from mind–body therapies on QoL domains among cancer survivors, the body of research is limited by poor quality RCT designs with heterogeneous outcomes. Richardson [33] discussed whether RCT designs, representing the research paradigm of reductionism, are an appropriate approach to study effects from complementary therapies, instead suggesting mixed methods designs involving both objective measures of effect and qualitative inquiry into the individual’s subjective experience as more suitable for complementary therapy research. The qualitatively generated information can be used to develop theoretical models that illustrate cancer patients’ determination process, as well as to generate new hypotheses that can be tested in meaningful quantitative studies. A meta-synthesis of 26 qualitative studies on cancer patients’ experience of using complementary therapies, including mind–body techniques, revealed that the patients experienced the complementary therapies as a niche of control in a time of uncertainty, contributing to the restoration of contact between body and mind, and to the development of social relationships [34].

One important finding was that the included reviews lacked dissemination of adherence rates from the mind–body trials, and gave little attention to adherence in the discussion sections. Exercise adherence is of great importance in interventions aiming at improvement of health and wellness outcomes, and poor adherence may negatively affect the outcome of complementary therapy interventions. Many breast cancer survivors face a decline in physical activity (PA) levels at the time of diagnosis, and face a huge challenge in exercising through treatment and survivorship according to the recommended PA level [35]. Interrelated factors such as treatment side effects, time constraints, and lack of support have been found to negatively affect exercise adherence [36].

Our overview identified a connection between positive QoL effects and amount and duration of yoga interventions. One study in the overview suggests that to have an impact on QoL, yoga should be performed for at least 90 days [18], while the more yoga classes completed, the more likely QoL will be enhanced [24]. Lally et al. [37] found great variation (range: 18–254 days, mean: 66 days) in how long it took for a health behavior to be performed automatically, and considered adopted by the individual. Raghavendra et al. [38] identified regularity of attendance at yoga classes to significantly predict patient QoL during chemotherapy treatment for breast cancer. Speed-Andrews et al. [39] studied determinants of Iyengar yoga class attendance among breast cancer survivors within the framework of the Theory of Planned Behavior (TPB), and identified the women’s intentions, self-efficacy and attitudes as significantly predicting yoga adherence. Similar results have been found in a recent meta-analysis [36] on predictors of exercise adherence among cancer populations, establishing intentions and perceived behavioral control as the most significant TPB constructs. The TPB can be a useful framework for future mind–body exercise interventions in cancer care and rehabilitation.

This overview study holds the potential to provide information on the usefulness of mind–body exercise in breast cancer care, based on the comparison of conclusions from multiple high-quality systematic reviews and meta-analyses [12]. It builds on conclusions from reviews of a total of 142 original mind–body exercise trials, including 123 RCTs. Nevertheless, limitations must be taken into consideration. Even though our literature search was comprehensively performed in multiple databases using Medical and CINAHL subject headings, and supplemented with hand-searching of reference lists, we might have missed relevant review articles, and publication bias cannot be ruled out. The search was performed in all five databases simultaneously. Searching the databases one by one could have allowed for a more accurate search. We limited our search to the search fields of title and abstract, which excludes articles lacking the search terms in the selected search fields. Overview studies are prone to overlap on included studies between the reviews and meta-analyses [28]. In the present overview, this well may be the case, especially concerning the meta-analyses of yoga studies, since Kelley and Kelley [9] included five of the studies in their overview study. Although our review largely confirms the findings of Kelley and Kelley [9], the issue of overlap calls for caution in interpreting the results.

The quality of the overview analysis depends on consistency of research methods and reporting practice in the included articles [12]. Three of eleven included reviews reported from a mix of complementary therapies [15,16,21]. The complexity of therapies might compromise the differentiation between effects from yoga, tai chi chuan or qigong, and could potentially affect the conclusions of the overview study. Consequently, two full-text papers had to be removed from the analysis [40,41]. However, consistent reporting of findings in the remaining review articles enabled extraction of effects from the specific therapies of interest. 

Inconsistency of intervention designs represents a challenge in an overview synthesis, making it a challenge to produce coherent judgements [42]. Mind–body exercise styles in the included review studies showed great variation, and none of the reviews distinguished the change in effect size between the different yoga, tai chi chuan and qigong styles used in the trials. Although not interchangeable, the different mind–body styles share common elements of physical postures, movements, controlled breathing and meditation [43]. Also, a more consistent reporting on the intervention mode could aid conclusions on what types of mind–body exercise therapies could have the greatest impact on quality of life in breast cancer survivors [23].

Quality assessment by the AMSTAR revealed that seven of the eleven included reviews yielded a high-quality score of 8–9.9 points. Nevertheless, major limitations concern three of the quality domains (i.e., domains 4, 5 and 10), which achieved fewer than five of eleven points across studies. They all address issues of publications bias, likely to complicate the conclusions of the reviews, related to overestimation of effects [44]. Although a comprehensive search for literature should be applied (ref. AMSTAR domain 4), allowing for inclusion of reports of any publication type (i.e., grey literature), it is difficult to predict the value of including such publications in a systematic review [42]. Only the study of Cramer et al. [23] used visual interpretation of funnel plots in assessing publication bias (ref. AMSTAR domain 10). This method is widely used but can be criticized for being too subjective, raising questions about its utility [10]. AMSTAR domain (5) yielded zero points across included reviews, mainly due to the fact that this domain holds a certain similarity to domain (6), and may appear difficult to apply in its current state. Future use of the AMSTAR could benefit from domain (5) solely targeting excluded studies. In all of the included meta-analyses, significant heterogeneity was detected and debated [15,18,19,20,22,23]. Sources of heterogeneity were variability of QoL outcome measurements, intervention modes and timing and diverse patient demographics, which might compromise the conclusions on effects from mind–body therapies. 

## 5. Conclusions

Distribution and use of complementary therapies is increasing in cancer populations, and several of these are offered to cancer survivors. The literature covering the last decade shows that among the mind–body therapies of yoga, tai chi chuan and qigong, the evidence is strong that yoga might aid women with breast cancer to improve their QoL. In particular, yoga seems to have a positive effect on breast cancer survivors’ experiences of psychological and social well-being, could help to restore their body-image and self-esteem, and may contribute in returning to their pre-diagnosis daily life. This knowledge has the potential to inform cancer care practice and rehabilitation in terms of administration and delivery of mind–body exercise. Tai chi chuan and qigong exercise holds potential for improvements of QoL in breast cancer survivors, but more research is warranted to synthesize evidence of benefits which can inform cancer survivors and cancer care.

Future knowledge summaries should include randomized controlled trials of high quality, which enable synthesizing results in meta-analyses and evaluation of heterogeneity. Synthesizing findings from reviews based on qualitative research is recommended, because such an approach may produce valuable information on patient’s experiences of benefits from complementary therapies that is lost in pure quantitative summations.

## Figures and Tables

**Table 1:** Literature search strategy.

Search	Database	Search Terms	Limitations	Identified Titles
#1	EBSCO *	Breast cancer	January 2007–March 2017 Abstract English language	210,484
#2	EBSCO	Review	January 2007–March 2017 Abstract English language	2,233,944
#3	EBSCO	Quality of life	January 2007–March 2017 Abstract English language	414,926
#4	EBSCO	#1 AND #2 AND #3	January 2007–March 2017 Abstract English language	1319
#5	EBSCO	Yoga	January 2007–March 2017 Abstract English language	16,772
#6	EBSCO	Tai chi	January 2007–March 2017 Abstract English language	3269
#7	EBSCO	Qigong	January 2007–March 2017 Abstract English language	1201
#8	EBSCO	#5 OR #6 OR #7	January 2007–March 2017 Abstract English language	20,514
#9	EBSCO	#4 AND #8	January 2007–March 2017 Abstract English language	37

* EBSCO—Elton B. Stephens Company.

**Table 2:** Characteristics of included review studies.

Author (Year)	Cancer Site Age Treatment Status	Mind–Body Intervention	Outcome	Study Designs Number of Studies (N)	Main Results
Bleakley & Stinson (2011) [21]	Breast 18–70 During and after treatment	Yoga Visualization Therapeutic massage Guided imagery Relaxation	Global QoL	RCT N = 4 Non-RCT N = 3 Cross-sectional N = 1	Four out of eight studies concluded that body–mind therapies had a positive effect on patients’ QoL.
Buffart et al. (2012) ** [22]	Breast 44–63 During and after treatment	Yoga	Global QoL HRQoL	RCT N = 13	Yoga had a large beneficial effect on anxiety and depression, a moderate but significant effect on HRQoL and fatigue and no significant effects on physical function.
Cramer et al. (2012) ** [23]	Breast 44–63 During and after treatment	Yoga	Global QoL	RCT N = 10	Yoga had a short-term effect of moderate size on global QoL, and a short-term effect of small size on functional, social and spiritual quality of life. No evidence for longer-term effects of yoga in breast cancer patients and survivors were found.
Harder et al. (2012) * [24]	Breast 45-63 During and after treatment	Yoga	Global QoL	RCT N = 18	Yoga had moderate-to-large effects on global and emotional QoL. The effect of yoga was greatest among patients with most yoga classes.
Lee et al. (2007) * [17]	Breast 30–78 During treatment	Tai Chi Chuan	HRQoL	RCT N = 3 Non-RCT N = 1	Positive effect of tai chi chuan was detected in self-esteem.
Levine & Balk (2012) [25]	Breast n.r. During and after treatment	Yoga	Global QoL	RCT N = 8 Non-RCT N = 2	Positive effects on global QoL, on symptoms of illness and treatment side-effects. Reduced fatigue and improved sleep quality, and positive results in physical, social and functional adaptation.
Pan et al. (2015) ** [19]	Breast 49–65 During treatment	Tai Chi Chuan	Global QoL HRQoL	RCT N = 9	Tai chi chuan led to no substantial improvement in HRQoL, or global HRQoL.
Pan et al. (2017) ** [18]	Breast 30–70 During treatment	Yoga	HRQoL	RCT N = 16	Yoga significantly improved overall HRQoL but had limited effect on physical well-being. Intervention duration >3 months showed better QoL.
Shneerson et al. (2013) ** [15]	Breast ≥18 After treatment	Yoga Qigong Meditation Mindfulness	Global QoL	RCT N = 13	Yoga had significant effects on global and mental QoL, but not on physical QoL. One study found significant effect of qigong on all QoL domains.
Stan et al. (2012) [16]	Breast n.r. After treatment	Yoga Tai Chi Chuan Qigong	Global QoL	RCT N = 23 Non-RCT N = 2 One-arm pilot N = 10	Yoga had statistically significant or trending toward significant effects. No strong evidence was found for effect from tai chi chuan and qigong.
Zhang et al. (2012) ** [20]	Breast ≥30 years old During treatment	Yoga	Global QoL	RCT N = 6	Yoga resulted in a statistically significant effect.

QoL—quality of life; RCT—randomized, controlled trial; HRQoL—health related quality of life; n.r.—not reported, *—systematic review; **—meta-analysis/systematic review.

**Table 3:** Quality assessment of included reviews.

Domain ^1^	1	2	3	4	5	6	7	8	9	10	11	AMSTAR Score ^4^
Study												
Bleakley & Stinson (2011) [21]	Yes	No	Yes	No	No	Yes	Yes	Yes	No	No	No	5
Buffart et al. (2012) [22]	Yes	Yes	Yes	No	No	Yes	Yes	Yes	Yes	No	Yes	8
Cramer et al. (2012) [23]	Yes	Yes	Yes	CA ^2^	No	Yes	Yes	Yes	Yes	Yes	Yes	9
Harder et al. (2012) [24]	Yes	Yes	Yes	Yes	No	Yes	Yes	Yes	No	No	Yes	8
Lee et al. (2007) [17]	Yes	Yes	Yes	Yes	No	Yes	Yes	No	No	No	No	6
Levine et al. (2012) [25]	Yes	CA	Yes	No	No	No	No	No	Yes	No	No	3
Pan et al. (2015) [19]	Yes	Yes	Yes	CA	No	Yes	Yes	Yes	Yes	No	Yes	8
Pan et al. (2017) [18]	Yes	Yes	Yes	CA	No	Yes	Yes	Yes	Yes	No	Yes	8
Shneerson et al. (2013) [15]	Yes	Yes	Yes	No	No	Yes	Yes	Yes	Yes	Yes	Yes	9
Stan et al. (2012) [16]	Yes	Yes	Yes	Yes	No	Yes	No	Yes	No	No	No	6
Zhang et al. (2012) [20]	Yes	Yes	Yes	No	No	Yes	Yes	Yes	Yes	NA ^3^	Yes	8
Domain total score across studies	11	9	11	3	0	10	9	9	7	2	7	

^1^ Quality assessment domains: 1 was an ‘a priori’ design provided; 2 was there duplicate study selection and data extraction; 3 was a comprehensive literature search performed; 4 was the status of publication (i.e., grey literature) used as an inclusion criteria; 5 was a list of studies (included and excluded) provided; 6 was characteristics of the included studies provided; 7 was the scientific quality of the included studies assessed and documented; 8 was the scientific quality of the included studies used appropriately in formulating conclusions; 9 was the methods used to combine the findings of studies appropriate; 10 was the likelihood of publication bias assessed; 11 was potential conflicts of interest included. ^2^ CA: Can’t answer. ^3^ NA: Not applicable. ^4^ A MeaSurement Tool to Assess systematic Reviews (AMSTAR) score: very high ≥10, high: 8–9.9, medium: 4–7.9, low: 0–3.9.
